# Experiment study on the corrosion resistance of the surface metamorphic layer of grinding

**DOI:** 10.1038/s41598-021-03493-4

**Published:** 2021-12-14

**Authors:** Xiaoliang Shi, Shichao Xiu, Xiao Liu

**Affiliations:** 1 School of Mechanical Engineering and Automation, Northeastern University, Shenyang, 110819 China; 2 Northeastern University, Shenyang, 110819 China

**Keywords:** Mechanical engineering, Materials science, Electrochemistry

## Abstract

Workpiece will face corrosive problems during its application after the manufacturing process. As the common final process, grinding can generate special metamorphic layer on the surface of workpiece and change the initial corrosion resistance of workpiece. In order to study the corrosion resistance of workpiece after grinding process, the paper carries on combining experiment of grinding and electrochemical corrosion. The characteristic of corrosion resistance of grinding is revealed based on the association of grinding mechanism and electrochemical theory. The corrosion potential of workpiece after grinding is higher than matrix, which shows the grinding surface is difficult to begin to corrode. Electrochemical impedance spectroscopy (EIS) shows the grinding surface has large phase angle, impedance and capacitance characteristic because the metamorphic layer of grinding has good obstructive ability. They reveal that grinding improves the surface corrosion resistance of workpiece. Then the mechanism of the corrosion resistance of grinding is revealed. The special grain boundary formed in grinding with much C element, large clusters and complex shape prolongs the corrosion channel, which reduces the corrosive speed. While, the sensitive hardening structure generated in grinding hardening with much free energy is easy to form the corrosion cell, which will accelerate the corrosion.

## Introduction

Grinding is always the final procedure in the manufacturing process, which will decide the final characteristic of surface layer and its surface integrity of the workpiece. So the grinding has a key meaning to technology level of the modern equipment manufacturing^1–3^. Before the grinding process, the workpiece is always machined by all kinds of cutting process, such as the turning, milling, boring and planning. Therefore, the grinding, on the one hand, needs to lower the machining errors of the former process and increase the machining precision of workpiece, then needs to obtain the high quality of surface integrity in the surface layer during the process on the other hand^4–7^. Grinding uses the abradant to remove the materials of workpiece. The grinding wheel has a large linear velocity and the strain rate of the processed material is large^8,9^. Combining with the cutting effect with large numbers of abrasive grain, large grinding force and high grinding temperature are generated during the grinding process, adding the factors of mechanical vibration. What’s more, the impacting effect by the abrasive grain also leads to the plastic deformation of the materials of surface workpiece, resulting the generation of the residual stress and the work hardening on the surface of workpiece. Then the surface of workpiece produces a special layer which is different from the inner part due to the coupling function of all the complex factors above. It can be called as the metamorphic layer and will determine the working performance of workpiece after grinding^10–13^, such as the tribology features, contacting fatigue properties and the corrosion resistance which is the target of the paper. The relation between corrosion resistance and grinding is very important but always neglected. Grinding always served as the final process, and workpiece will face all kinds of working environment after grinding. The corrosion resistance after grinding determines whether the workpiece can perform well in its place as it will face many corrosive problems during its working life inevitably. And the grinding will have influence on the corrosion resistance of workpiece after the manufacturing process because it will generate special metamorphic layer on the surface of workpiece, which changes the important factors of corrosion resistance. Therefore, the corrosion resistance of workpiece after grinding is important to the manufacturing industry.

At present, scholars have done some researches on the corrosion resistance related to grinding. Ohmoril et al. studied the corrosion resistance of metallic biomaterials using a new electrical grinding technique^14^. The electrical grinding method improves oxide formation on the finished surfaces, thereby realizing finished surfaces with extremely thick and stable oxide layers. Burkert et al. studied the influence of grinding treatment of stainless steels on the corrosion behavior^15^. They found the variation of different grinding conditions have a substantial influence on the corrosion resistance and different corrosion susceptibility could be proven by means of electrochemical noise measurements. Wang et al. investigated the surface corrosion behavior of inconel 718 after robotic belt grinding^16^. They found the corrosion resistance of the specimen surface improves remarkably with the decrease of abrasive particle size. Zhou et al. studied the influence of surface grinding on corrosion behavior of ferritic stainless steels in boiling magnesium chloride solution^17^. They carried on corrosion tests in boiling magnesium chloride solution. No macro-cracking was found on any specimen after exposure even at high calculated applied loads. Panadda and Hathaipat explored the novel application of electroplating technique and surface grinding to enhance the coating's ability to resist chemical corrosions further^18^. They found the corrosion behavior could be improved by smoothening of the coating surface prior to the plating process.

The present researches mainly concentrate on the relations of corrosion resistance with the surface roughness and residual stress after grinding, or the direct effect of grinding parameters on the corrosion resistance in some special grinding methods. While, seldom research is concentrated on the corrosion resistance of the actual surface layer of workpiece after grinding. The grinding process generates special microstructure on the surface of workpiece, in return for this, the special microstructure reveals special outer property. The microstructure of metamorphic layer under the microscale has a great influence on the outer mechanical property of material. The boundary of grain after grinding may have influence on the corrosion channel. The diversity of electrochemistry between phase of grinding and mother phase may also affect the corrosion course of grinding surface. Therefore, it seems to be more intrinsic to reveal the mechanism of the corrosion resistance after grinding from the view of the microstructure in the metamorphic after grinding. The paper will carry on study on the aspect based on the grinding and corrosion experiment and then reveal the characteristic and mechanism of corrosion resistance after grinding in more nature.

## Experiment

### Grinding experiment

**Figure 1:**
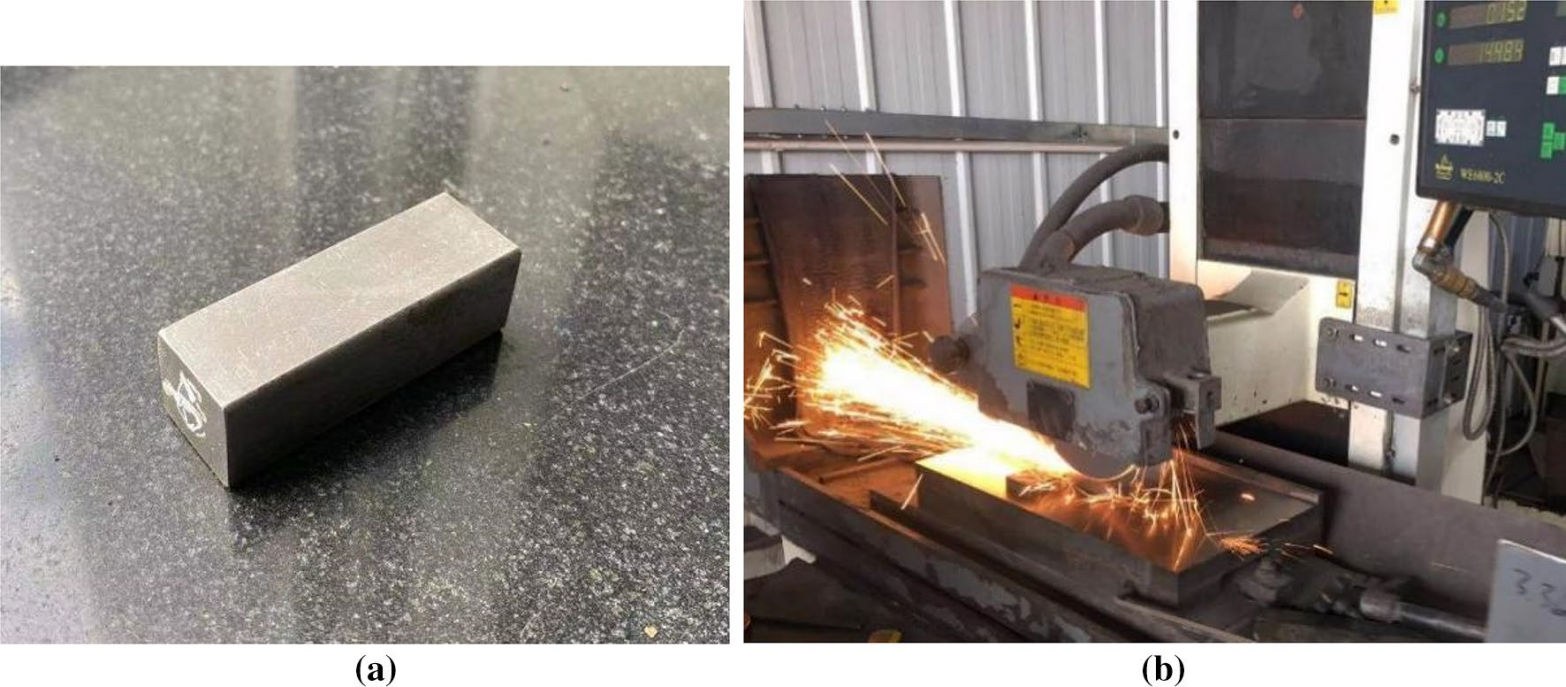
Grinding experiment: (**a**) experiment workpiece, (**b**) process of grinding experiment.

The material of grinding experiment is 45 steel. Its composition is shown in Table 1. It is a common material in the mechanical processing. The experiment workpiece is processed as 45 mm × 15 mm × 15 mm which is shown in Fig. 1a. The grinding width is the width of experiment workpiece which is 15 mm.

**Table 1:** Chemical composition of 45 steel.

C	Si	Mn	Cr
0.37–0.45%	0.17–0.37%	0.50–0.80%	0.80–1.10%

WE6800-ZC flat grinder is taken as the experiment equipment. White-corundum grinding wheel made of ceramic adhesive is chosen as the grinding wheel. Its grain size is F46 and diameter is 250 mm.

Down grinding has better effect on producing the metamorphic layer. So the experiment takes down grinding. Coolant liquid is not taken to obtain processing layer with more obvious features of grinding. The grinding depth chooses 50 μm which belongs to common grinding and 100 μm which belongs to grinding hardening. The feeding speed of workpiece is 0.02 m/s. The experiment process is shown in Fig. 1b.

### Observation of surface metamorphic layer

The surface metamorphic layer after grinding is observed first to assist the study on its corrosion resistance. The workpiece after grinding is cut by wire-electrode cutting machine to observe its microstructure of section. The sections are polished by the abrasive paper from small mark to big mark along with interlaced direction first. Then they are polished on the polishing machine with diamond polishing paste whose granularity is 20. After the polishing, the section of workpiece is corroded by the nitric acid with 4% concentration in alcohol to reveal its grain boundary.

After the procedures above, the preprocessing of workpiece is completed to go on further observation. The OLYMPUS GX71 metalloscope is used to observe the metallographic of surface layer of workpiece after grinding and grain boundary preliminarily. The SSX-550 scanning electron microscope (SEM) is used to observe the microstructure of workpiece surface further. The distribution of element of microstructure is detected by the JXA-8530F electronic probe tester.

### Electrochemical experiment

**Figure 2:**
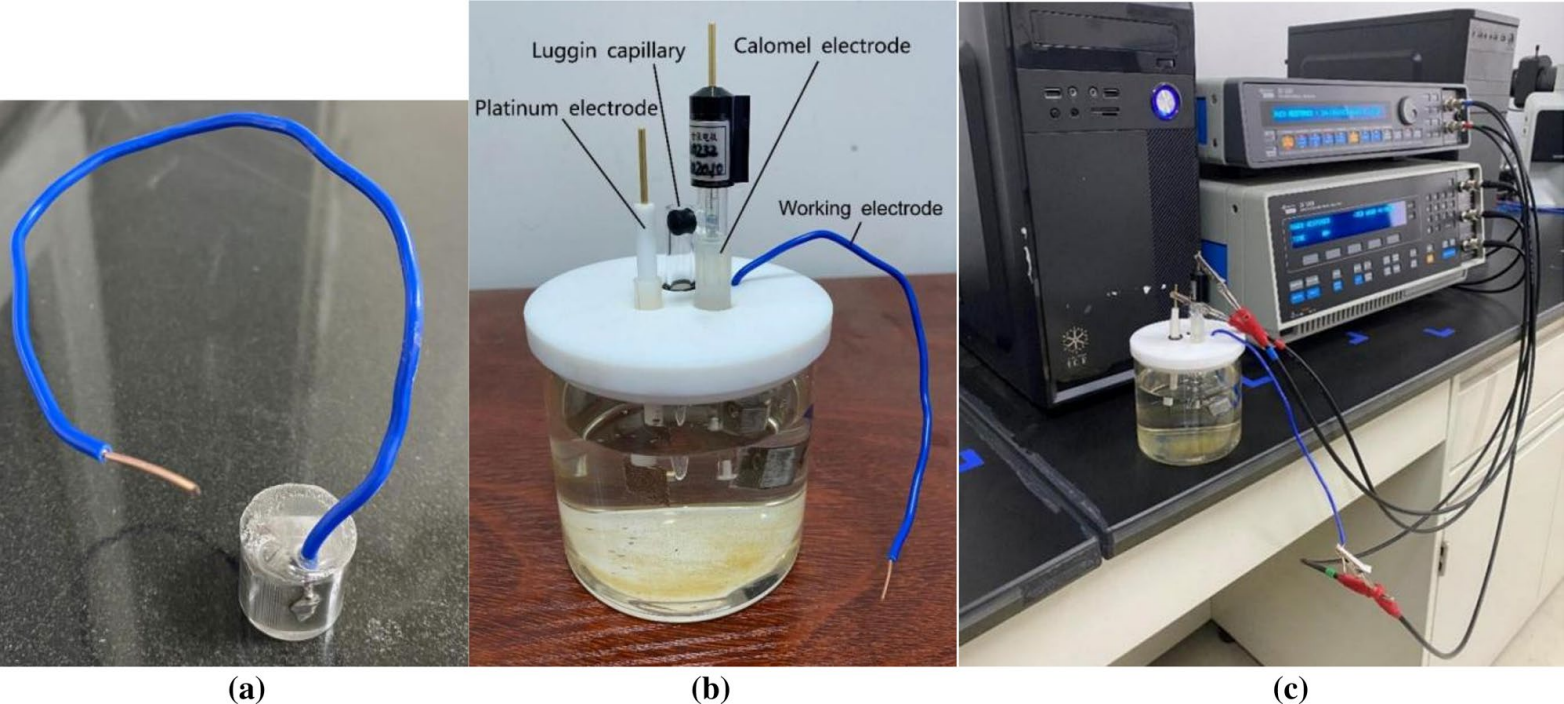
Electrochemical corrosion experiment: (**a**) processing of workpiece, (**b**) whole three-electrode system, (**c**) process of the electrochemical corrosion experiment.

In order to study the corrosion resistance of grinding workpiece and accelerate the corrosion process, the paper takes the three-electrode system to get on electrochemical research on the workpiece after grinding. The electrochemical experiment consists of working electrode, counter electrode and reference electrode. The three electrodes form two loops. One loop consists of working electrode and reference electrode which tests the electrochemical reaction of working electrode. The other loop consists of working electrode and counter electrode which plays a role of electron transport. SI1260 electrochemical workstation is used to carry on the experiment. Through comprehensive consideration of the common experiment of other researchers, the 4% NaCl is chosen as the corrosive liquid. It is a representative corrosive liquid which can simulate the corrosion of working life. The workpiece serves as the working electrode. The remaining part of the same workpiece is cut to the size of 10 mm × 15 mm × 15 mm by the wire-electrode cutting and welded with an electric wire. The machining surface of workpiece is exposed to the corrosive solution and the other surface is covered with thermosetting resin. The workpiece after all the pretreatment is shown in Fig. 2a. The exposed size is 15 mm × 10 mm. The platinum electrode is chosen as the counter electrode. The size of the platinum sheet is 15 mm × 15 mm. Its area is larger than the exposed workpiece to guarantee the uniform of the whole electrochemical reaction. The calomel electrode is chosen as the reference electrode. What more, the Luggin capillary is used to eliminate the overpotential of resistance. It can reduce the ohmic potential drop of solution. So it makes for the measurement and controlling of the potential of working electrode accurately without influencing its distribution of the electric field. The whole parameters are listed in Table 2. The whole test system is shown in Fig. 2b. The process of the electrochemical corrosion experiment is shown is Fig. 2c. The electrochemical parameters including the open circuit potential, the polarization curve and the electrochemical impedance (EIS) are measured by the SI1260 electrochemical workstation automatically. The results are used to the analyze the corrosion property of workpiece after grinding.

**Table 2:** The selection of electrochemical experiment.

Item	Content
Corrosive liquid	4% NaCl
Working electrode	Workpiece
Counter electrode	Platinum electrode
Reference electrode	Calomel electrode
Auxiliary means	Luggin capillary
Range of voltage	− 0.75 V to 0.5 V
Range of frequency	10^−1^ Hz to 10^5^ Hz

### Observation of the topography after corrosion

The surface topography of workpiece after corrosion can show some characteristics of the corrosion process. The 3D surface topography of grinding workpiece after electrochemical corrosion is observed by LEICA DVM6 3D microscope with super wide depth of field. The magnification times is 1000. The step number of composition of depth of field is 20. Then the corrosive topography will be analyzed to assist the research on the corrosion characteristics of grinding.

## Analysis of the experiment result

### Analysis of metamorphic layer of grinding

**Figure 3:**
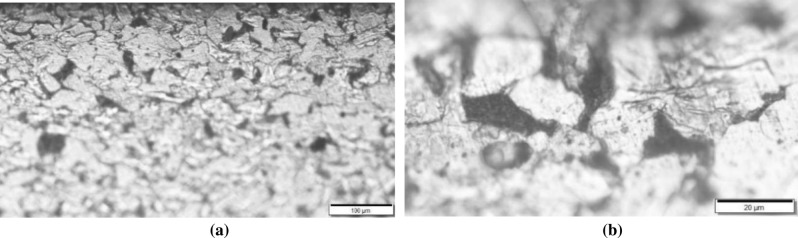
Metallographic of workpiece after grinding: (**a**) magnification × 100, (**b**) magnification × 500.

The characteristics of metamorphic layer of grinding are analyzed first to provide basis for the study on its corrosion resistance. Figure 3 is the metallographic of cross section of workpiece after grinding with different magnification times. It can be seen from Fig. 3a that the grain is stretched and its boundary orientation is along with the manufacturing direction because the grinding effect influences the inner layer and oppresses the grain boundary. Figure 3b shows partial phase transformation happens due to the grinding heat generated in the grinding process. Microstructure mainly contains pearlite and ferrite. It also can be seen that some bainite phase separates out.

**Figure 4:**
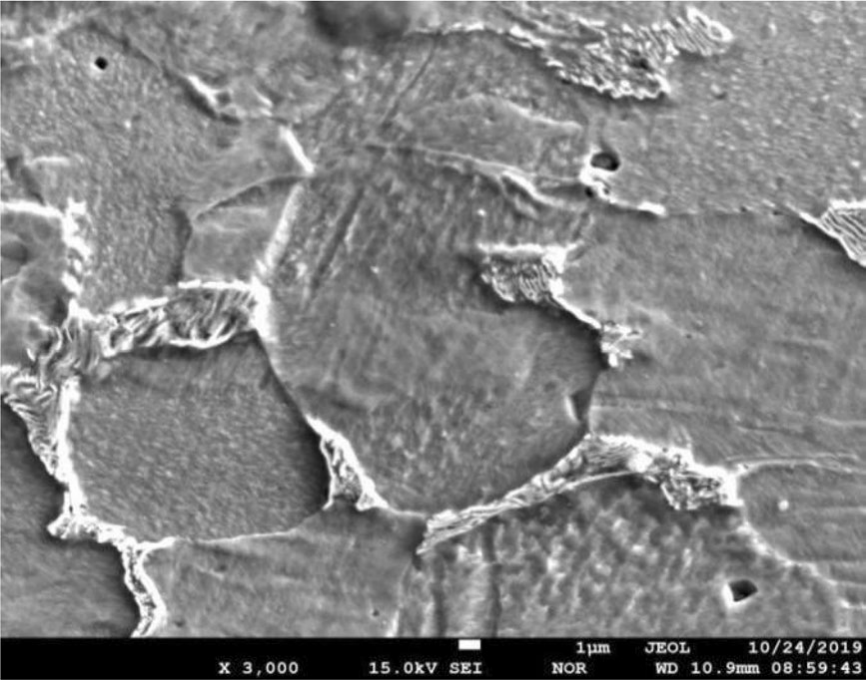
The characteristic of grain boundary of grinding by SEM.

Figure 4 shows the SEM of the metamorphic layer of grinding. The grain boundary of workpiece after grinding is demonstrated in detail. It can be seen that the grain has large clusters and its boundary has complex shape. The microstructure is uniform. Both high angle grain boundary and low angle grain boundary are generated. Some area generates special trigeminal boundary and substructure among the grain clusters. Some twin grain also forms on the metamorphic layer of workpiece.

During the grinding process, the grinding wheel rotates in high speed, and the abrasive grain collides and cuts with the surface of the workpiece. The collision causes large plastic deformation and temperature in the contacting area between the workpiece and abrasive grain. The dislocation and the flow stress increase. Then the crystal lattice distorts and the grain is stretched or even broken and reveals the characteristics as is shown in Fig. 4.

**Figure 5:**
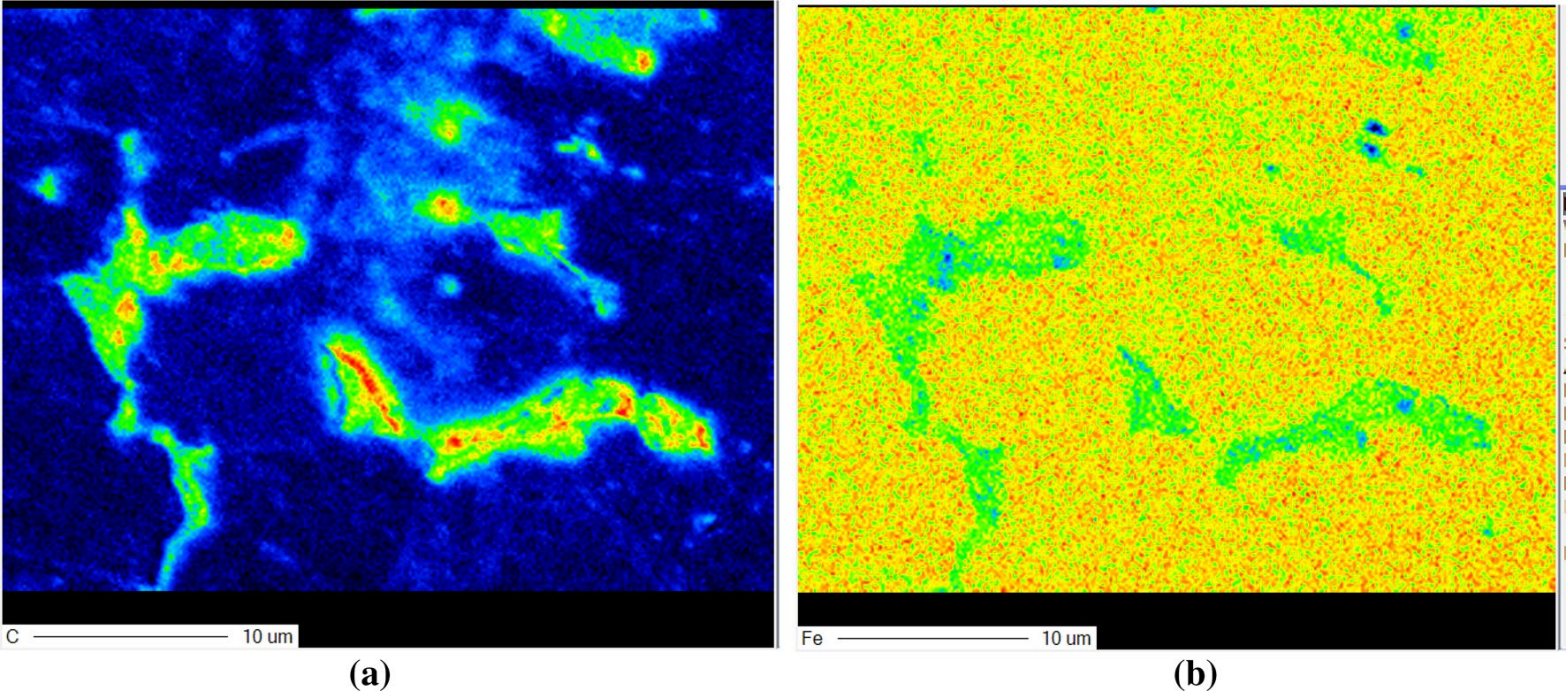
The plane distribution of element of the grain boundary of grinding: (**a**) C element, (**b**) Fe element.

The distribution of element of the corresponding grain boundary which is detected by electronic probe technology is shown in Fig. 5. It can be seen that the location of boundary has more C element than the inner area of grain and the Fe element distributes more in the inner area of grain. It shows that the carbide separates out in the grain boundary gradually and gathers here. And it may form some carbide or the microstructure with much C element. The feature of grain boundary of workpiece after grinding can influence the external characteristic of material.

### Analysis of polarization curve

**Figure 6:**
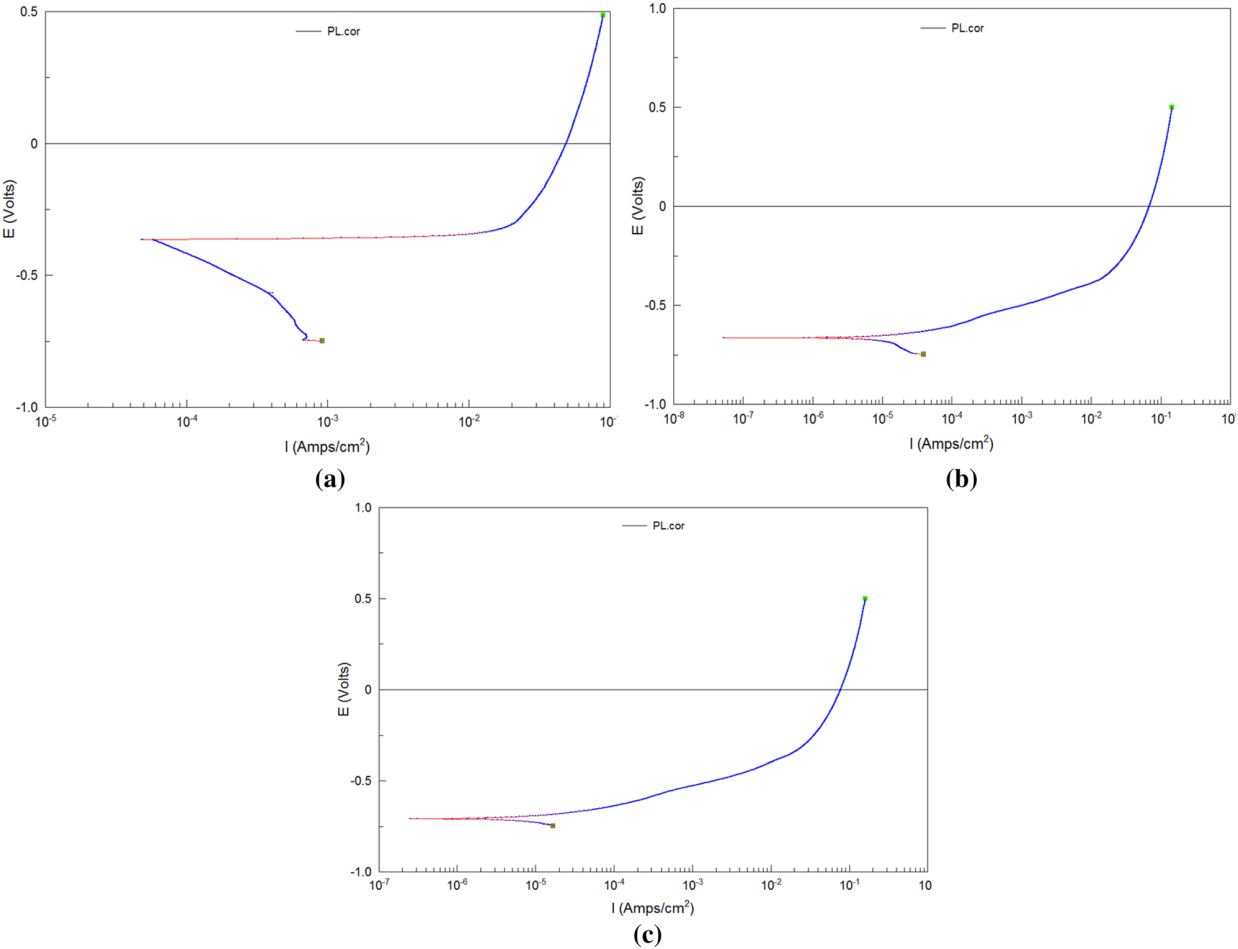
Potentiodynamic polarization curve of the corrosion process: (**a**) grinding (*a*_p_ = 50 μm), (**b**) matrix, (**c**) grinding hardening (*a*_p_ = 100 μm).

Figure 6 is the potentiodynamic polarization curve of the corrosion process of grinding workpiece. It shows the variation of the corrosion potential and corrosion current during the corrosion process. The corrosion potential is a thermodynamics parameter of corrosion which reflects the difficult degree that the surface of metal begins to corrode. And the corrosion current is a dynamics parameter of corrosion which reflects the speed of the corrosion on the surface of metal^19^.

The polarization curve of common grinding with small depth of cut is analyzed first which is shown in Fig. 6a. At the initial stage of corrosion reaction which is the active area, the slope of the cathode segment of polarization curve is moderate. The speed of reaction isn’t very fast. And there is a certain resistance of reaction. It shows the characteristic boundary of surface layer of grinding delays the corrosion course at this time. As the reaction goes on, the slope of the cathode segment of polarization curve begins to turn small. The resistance of reaction becomes small and the speed of reaction accelerates. The characteristic boundary of surface layer of grinding may have been corroded at this time which plays the role of anti-corroding. And it comes into the easy-corrosive area. When it comes into the anode segment of polarization curve, it has no passivation area. The passivation area is an area in the polarization curve where the reaction has obvious stagnate. In the study, the polarization curve has no passivation area, which means the reaction of the state is happening continuously. It reflects that the corrosion is uniform in the corresponding area of the grinding surface layer. The reaction comes into the excessive passivation area directly. The slope becomes big. The resistance of reaction becomes large at the stage and the speed of reaction becomes slow. The corrosion potential of the surface layer of common grinding workpiece is about − 0.3 V on the whole in Fig. 6a.

Figure 6b is the potentiodynamic polarization curve of the matrix that is the metal surface which hasn’t been ground. It can be seen that the corrosion potential of workpiece before grinding is about − 0.7 V and is lower than the grinding surface compared with their polarization curve. It means the unprocessed workpiece is easier to begin to corrode. The slope of the cathode segment of polarization curve is larger. The resistance of reaction is small and the speed of reaction is large. It shows the common grinding improves the critical value that grinding workpiece begins to corrode on the whole.

Figure 6c is the potentiodynamic polarization curve of the grinding workpiece with large cutting depth which belongs to the range of grinding hardening. It can be seen that the corrosion potential and corrosion current of workpiece of grinding hardening are similar to the matrix, which reveals the corrosion resistance of grinding hardening is similar to the initial state of workpiece.

The grinding hardening can produce hardening effect on the surface of workpiece, which increases the surface hardness and abrasive resistance of workpiece and it doesn’t weaken the corrosion resistance of initial metal obviously. Therefore, the grinding hardening still has good comprehensive functional performance and mechanical property.

In the later stage of corrosion, the forms of polarization curve under different conditions tend to be the same. It shows the surface metamorphic layer of workpiece after grinding has been fully corroded. The corrosion has expended into the inner area of workpiece at the stage. And the inner microstructure of workpiece without being interfered by grinding is the same. So their polarization curves of the segment are similar to each other.

### Analysis of open circuit potential

**Figure 7:**
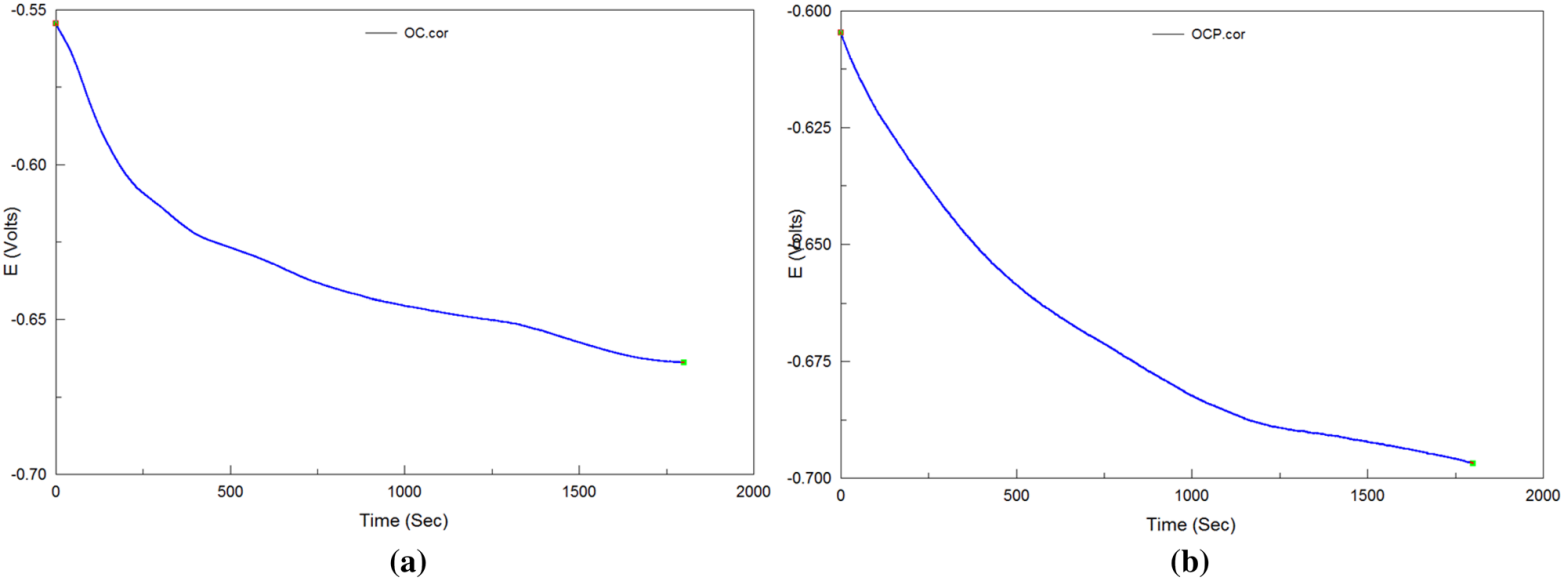
The open circuit potential: (**a**) grinding condition, (**b**) matrix.

Figure 7 is the open circuit potential of grinding. When the electrode contacts with solution, the surface of electrode will dissolve and absorb because the free energy of the electrode and solution are different, which causes the change of the surface potential of electrode. The open circuit potential represents the change and it reveals the tendency of corrosion. It tends to be the corrosion potential in principle. Because the polarization process will interfere the whole system, then the corrosion potential wanders and has difference with the open circuit potential. The fluctuation of open circuit potential of grinding is smaller than matrix, which shows the surface potential of electrode of grinding has little change.

### Analysis of EIS

**Figure 8:**
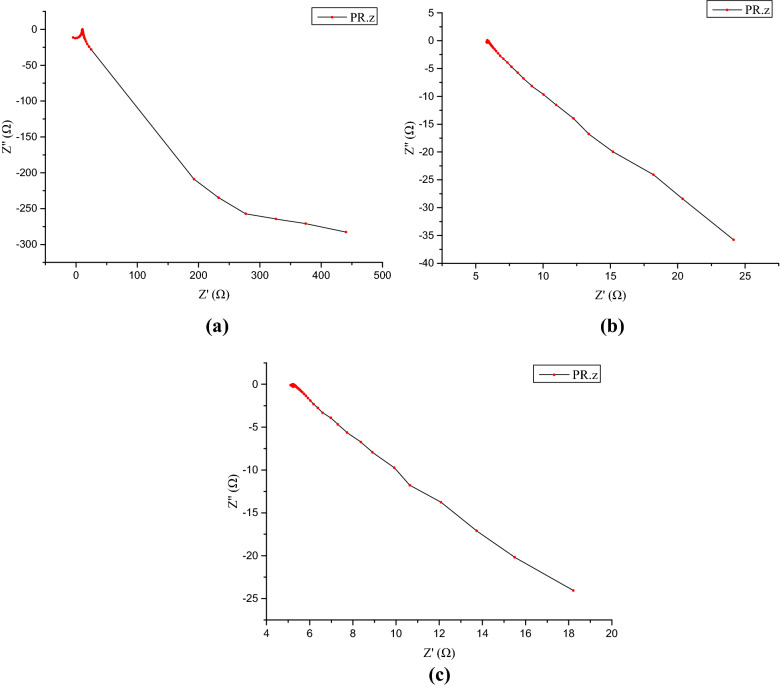
The Nuquist figure of grinding workpiece: (**a**) grinding (*a*_p_ = 50 μm), (**b**) matrix, (**c**) grinding hardening (*a*_p_ = 100 μm).

The EIS result contains the Nuquist figure and Bode figure. The Nuquist figure and Bode figure are different forms of expression for the impedance of grinding system. The Nuquist figure emphasizes the variation relation of real part and imaginary part. And the Bode figure pays attention to the relation between impedance modulus, phase angle and frequency. The Nuquist figure of the workpiece after grinding is shown in Fig. 8. The area which is close to the origin is the high frequency region and away from the origin is the low frequency region. And the Nuquist curve resembles an arc or a line with a radian, which is the characteristic of capacitive arc. Its diameter reflects the corrosion resistance of the manufacturing surface. The larger the diameter of the arc of capacitive reactance is, the larger the corrosion resistance of the surface is^20^. It can be seen that the range of Nuquist curve of the grinding in Fig. 8a has obvious larger order than the matrix in Fig. 8b and grinding hardening in Fig. 8c. If they are all regarded as arc, the diameter of the grinding surface is larger than the surface of unprocessed surface and the grinding hardening surface. It reveals the surface of grinding has better corrosion resistance.

**Figure 9:**
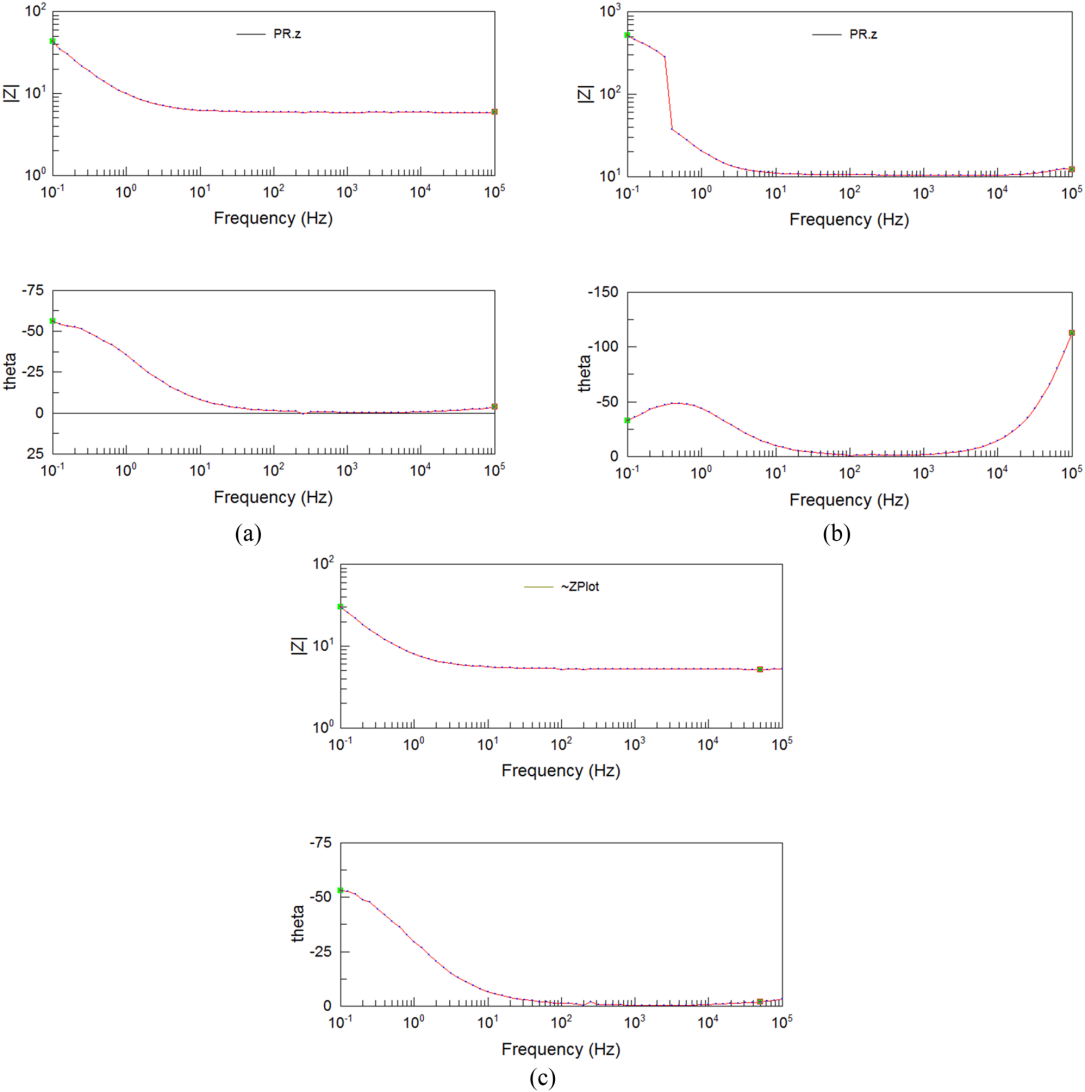
The Bode figure of grinding workpiece: (**a**) grinding (*a*_p_ = 50 μm), (**b**) matrix, (**c**) grinding hardening (*a*_p_ = 100 μm).

The Bode figure of the workpiece after grinding is shown in Fig. 9. It contains frequency-amplitude part which is in the upper half and the frequency-phase-angle part which is the in the bottom half. The frequency-amplitude of Bode can represent the arc of capacitive reactance of the Nuquist curve quantificationally. The high frequency region of the curve which is in the right side reflects the impedance of the solution. The impedance values of each curve are almost the same which are about 10 Ω. And the low frequency region of the curve which is in the left side of the curve reflects the impedance of the machined surface. The larger the impedance is, the better the corrosion resistance of the manufacturing surface is^19^. Figure 9a shows that the impedance of the grinding surface can reach over 600 Ω, which shows it has good corrosion resistance.

The frequency-phase-angle part shows that the biggest phase angles of grinding and grinding hardening distribute from about − 50° to − 60°. The biggest phase angle of matrix distributes from − 30° to − 50°. Compared with matrix, the grinding surface has larger range of phase angle, which reveals the grinding surface has larger capacitance characteristics, and it has better corrosion resistance in larger range. The phase angle of grinding surface decreases slowly in the low frequency region, which shows that the passive film formed on the surface is stable and it is hard to break through. It has stable corrosion resistance. The time constant can reflect the influence of factors during the electrode reaction. According to the Bode figure, the time constant of the grinding surface moves to smaller value compared with matrix, which reflects the influence of surface metamorphic layer on electrode reaction is different and the surface metamorphic layer of grinding has good obstructive ability. It obstructs the Na^+^ and Cl^−^ from permeating into the inner layer of matrix through surface layer along with the solution.

### Analysis of topography after corrosion

**Figure 10:**
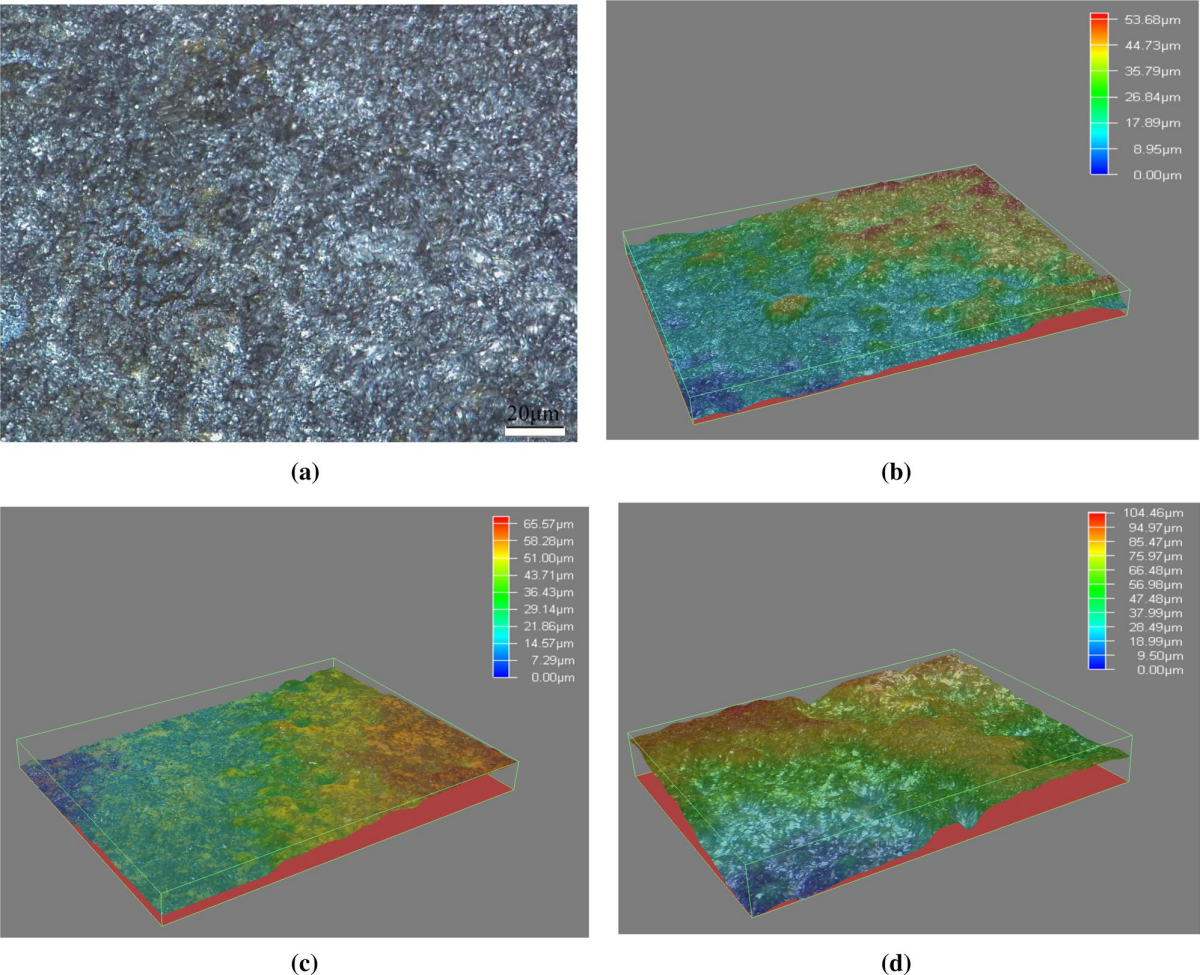
Surface topography of workpiece after corrosion: (**a**) 2D topography of workpiece after corrosion, (**b**) 3D topography of grinding, (**c**) 3D topography of grinding hardening, (**d**) 3D topography of matrix.

The surface topography of grinding workpiece after electrochemical corrosion is shown in Fig. 10. It can be seen from Fig. 10a the topography of workpiece is similar generally after the corrosion process. It can be seen that the surface of workpiece after grinding is no longer smooth after the corrosion process. Crystal distributes on the surface of workpiece in dispersive state. The crystal separates out during the corrosion process and heaps up on the surface of workpiece, which reveals the rough and uneven topography. The crystal with larger size is in the state of growth and the crystal with smaller size is in the state of nucleation. It also can be seen that some rust forms during the corrosion process and covers some area of the workpiece surface.

The 3D surface topography of grinding workpiece after electrochemical corrosion is shown in Fig. 10b–d. The characteristics of crystal and topography under different grinding conditions are compared. Figure 10b is the surface topography after corrosion of common grinding with small depth of cut. It can be seen that a little crystal falls off from surface and a few corrosion products heap up there. The corrosion groove marks are shallow. The maximum range between the peak and trough of topography is about 50 μm. The inner part is smooth and has no micro-crack.

Figure 10c is the surface topography after corrosion of grinding hardening with large depth of cut. Its corrosive topography is close to the common grinding with small depth of cut. The corrosion marks are shallow. The maximum range between the peak and trough of topography is about 60 μm. A little crystal falls off from surface as well. The whole corrosive topography is uniform.

Figure 10d is the surface topography after corrosion of the matrix. Large numbers of crystal fall off from surface and the fluctuation of topography is large, which are obvious different from the corrosive topography after grinding. The maximum range between the peak and trough of topography can reach about 100 μm. Its surface corrodes badly. Through the contrast of Fig. 10, it can be seen that the corrosive topography after grinding is more uniform with less corrosion, which accords with the previous analysis of polarization curve and EIS.

## Corrosion mechanism and discussion

### Whole reaction equation

In the electrochemistry corrosion of grinding workpiece, the whole three-electrode system forms the primary battery. Because the workpiece reacts in the NaCl solution, according to the experimental condition, it can be judged that the corrosion product is Fe(OH)_3_. The reaction process is as follows. The O_2_ is on the surface of NaCl solution and Fe is in the solution. This time, the electronic of the Fe can transfer to the O_2_ through the reaction.

The reaction equation is:

Cathode:

1 $${\text{Fe}} - 2{\text{e}}\left( {^{ - } } \right) = {\text{Fe}}\left( {^{2 + } } \right)$$

Anode:

2 $$2{\text{H}}\left( {^{ + } } \right) + 2{\text{e}}\left( {^{ - } } \right) = {\text{H}}_{2} \uparrow$$

Equation:

3 $${\text{Fe}} + 2{\text{H}}_{2} {\text{O}} = {\text{Fe}}({\text{OH}})_{2} + {\text{H}}_{2} \uparrow$$

where whether Fe is easy to lose its electronic in Eq. (1) is one of the factors of the corrosion resistance of grinding surface layer.

Then the Fe(OH)_2_ is oxidated by the O_2_ in the air and generates Fe(OH)_3_:

4 $$4{\text{Fe}}({\text{OH}})_{2} + {\text{O}}_{2} + 2{\text{H}}_{2} {\text{O}} = 4{\text{Fe}}({\text{OH}})_{3} \downarrow$$

During the process, the surface of grinding corrodes and generates rust.

### Influence of microstructure

As the previous analysis, grinding generates complex boundary of grain in the surface metamorphic layer of workpiece. And some boundary of grain has good corrosion resistance to the intergranular corrosion. The grain itself doesn’t corrode or just corrodes slightly. If the intergranular corrosion intends to happen, it generates in the boundary of grain and its adjacent area preferentially.

The special grain boundary in grain clusters of grinding can delay the corrosion from eroding into the inner clusters. The corrosion can’t extend across the inner clusters. The larger the size of the grain clusters is, and the more complex the shape is, the longer the corrosion channel is along which corrosive medium transmits^21^. When the corrosion reaction is happening, the concentration of corrosive medium at the leading end of corrosion channel will decrease due to the electrochemistry reaction. If the corrosion channel is long, the corrosive medium at the end of corrosion path will be hard to expand to the lending end. Then the corrosive speed at the leading end will slow down largely. Therefore, corrosion path is long in the workpiece after grinding with large-size grain clusters and it takes more time to make them fall off, which reduces the corrosive speed. Meanwhile, the special complex boundary after grinding has much C element and less Fe element which is analyzed in “Analysis of metamorphic layer of grinding”, then less Fe will lose its electronic and less reaction of Eq. (1) will happen. Then less Fe(^2+^) is provided as the initial element to go on corroding. Then the corrosion will not be severe in the situation. Therefore, grinding improves the corrosion resistance of the surface of workpiece as the polarization curve and EIS shows.

Besides the improving mechanism to the corrosion resistance which is the same as the common grinding, grinding hardening has the opposite effect which can weaken the corrosion resistance of workpiece. In grinding hardening, lots of grinding heat is generated and the surface material is heated quickly by the grinding heat. In the situation the hardening structure like martensite generates in the surface layer of material and hardening effect appears. During the process, the final microstructure and the initial microstructure have different specific volumes, which makes the material expand or shrink during the transformation and brings forth lots of inner stress. The structures with stress are unbalanced and very sensitive. They have large free energy. In the situation the reaction of Eq. (1) are easier to happen and the corrosion channel is easier to form, which results in the decrease of the corrosion resistance of the surface layer of grinding hardening. What’s more, the process also causes the supersaturated C element to precipitate as the carbide. The carbide will make up the corrosion cell with the ferrite of the mother phase^22^. The carbide serves as the cathode and the ferrite of the mother phase serves as the anode, which will accelerate the corrosion of the grinding hardening layer. The corrosion resistance of grinding hardening is the comprehensive function of two opposite mechanisms. Therefore, the corrosion resistance of grinding hardening is weaker compared with grinding. And it is close to matrix, which shows some index is better than matrix and some is worse.

### Influence of corrosion products

Besides the influence of the surface metamorphic layer of grinding, the protection for the surface by the corrosion products will also influence the corrosion process. During the corrosion process, lots of corrosion products are generated which mainly consist of the crystal with different shapes. The crystal heaps up on the surface of workpiece and forms the corrosive topography. The corrosive topography of common grinding and grinding hardening in Fig. 10 reveals that the corrosion products cover most of the surface of workpiece after the electrochemical corrosion. The corrosion products are liable to bonding to the remaining metamorphic layer during the corrosion process because the reaction in the metamorphic layer is slow and uniform and the corrosion products are easy to heap up on the basis. Moreover, the remaining microstructure with complex shape is more likely to absorb the corrosion products. Then the size of crystal grows continuously and its structure densifies. The dense products can prevent the corrosive ion from invading into the inner layer additionally^23^. Therefore, the corrosion products improve the resistant ability to the corrosive ion in the solution besides the corrosion resistance of grinding surface layer itself.

The surface of matrix is covered with less corrosion products after the corrosion process and they distribute unevenly. It reveals that corrosion products are not liable to bonding to the Fe matrix and they don’t heap up on the surface of workpiece efficiently. Then part of matrix continues to expose to the corrosive solution, which worsen the corrosion resistance. At this moment, the corrosive ion of the solution can pass the interstice of the corrosion products and go on corroding with the Fe^2+^ which is decomposed from the Fe matrix. Then the corrosion intensifies. Therefore, the surface matrix has a high level of corrosion after the electrochemical corrosion.

## Conclusions

The workpiece surface of grinding has high corrosion potential and is difficult to begin to corrode. The fluctuation of open circuit potential of grinding is small, and its surface potential of electrode changes little. The grinding surface has large phase angle, impedance and capacitance characteristic because the surface passive film and metamorphic layer of grinding has good obstructive ability. They reveal the surface of grinding has good corrosion resistance and it has corrosion resistance in larger range. The corrosion resistance of grinding hardening is similar to matrix.

The special grain boundary formed in grinding with much C element, large clusters and complex shape prolongs the corrosion channel and weakens the corrosion inducement, which reduces the corrosive speed. The sensitive hardening structure generated in grinding hardening has much free energy and is easy to form the corrosion cell, which will accelerate the corrosion.

## References

[1] Singh N, Dar AA, Kumar A (2018). A simple and efficient approach for the synthesis of 1,3-oxazolidines from beta-amino alcohols using grinding technique. ChemistrySelect.

[2] Li HS, Niu S, Zhang QL, Fu SX, Qu NS (2017). Investigation of material removal in inner-jet electrochemical grinding of GH4169 alloy. Sci. Rep..

[3] Thanedar A, Dongre GG, Singh R, Joshi SS (2017). Surface integrity investigation including grinding burns using Barkhausen noise (BNA). J. Manuf. Process..

[4] Wu YT, Zhang L, Ge PQ, Gao YF (2018). Experimental study of rectangular groove texture in the surface of photovoltaic silicon with diamond coated micro-milling tools. Mater. Sci. Semicond. Proc..

[5] Ma ZK, Perry L, Li Q, Yang XY (2019). Morphological changes in starch grains after dehusking and grinding with stone tools. Sci. Rep..

[6] Mao C, Liang C, Zhang YC, Zhang MJ, Hu YL, Bi ZM (2017). Grinding characteristics of cBN-WC-10Co composites. Ceram. Int..

[7] Dimaki AV, Shilko EV, Dudkin IV, Psakhie SG, Popov VL (2020). Role of adhesion stress in controlling transition between plastic, grinding and breakaway regimes of adhesive wear. Sci. Rep..

[8] Mao C, Ren YH, Gan HY, Zhang MJ, Zhang J, Tang K (2015). Microstructure and mechanical properties of CBN-WC-Co composites used for cutting tools. Int. J. Adv. Manuf. Technol..

[9] Yang M, Li CH, Zhang YB, Jia DZ, Li RZ, Hou YL, Cao HJ (2019). Effect of friction coefficient on chip thickness models in ductile-regime grinding of zirconia ceramics. Int. J. Adv. Manuf. Technol..

[10] Zhang MJ, Tan Y, Zhou FJ, Mao C, Xie ZZ, Li CH (2017). Analysis of flow field in cutting zone for spiral orderly distributed fiber tool. Int. J. Adv. Manuf. Technol..

[11] Zhang XH, Jiang RY, Li C (2021). Experimental evaluation of the lubrication performance of MoS_2_/TiO_2_ nanoparticles for diamond wheel bond in silicon carbide ceramic grinding. Int. J. Adv. Manuf. Technol..

[12] Zhang XH, Wang ZR, Shi ZY (2020). Improved grinding performance of zirconia ceramic using an innovative biomimetic fractal-branched grinding wheel inspired by leaf vein. Ceram. Int..

[13] Huang XM, Ren YH, Zheng B, Deng ZH, Zhou ZX (2016). Experiment research on grind-hardening of AISI5140 steel based on thermal compensation. J. Mech. Sci. Technol..

[14] Ohmori H, Katahira K, Nagata J, Mizutani M, Komotori J (2002). Improvement of corrosion resistance in metallic biomaterials using a new electrical grinding technique. CIRP Ann. Manuf. Technol..

[15] Burkert A, Schilling K, Heyn A (2004). The influence of grinding treatment of stainless steels on the corrosion behaviour. Mater. Corros..

[16] Wang JW, Xu JJ, Zhang XQ, Ren XK, Song XF, Chen XQ (2018). An investigation of surface corrosion behavior of Inconel 718 after robotic belt grinding. Materials.

[17] Zhou N, Pettersson R, Schonning M, Peng RL (2018). Influence of surface grinding on corrosion behavior of ferritic stainless steels in boiling magnesium chloride solution. Mater. Corros..

[18] Panadda N, Hathaipat K (2006). Improved corrosion resistance of thermally sprayed coating via surface grinding and electroplating techniques. Surf. Coat. Technol..

[19] Xu N, Sarkar DK, Chen XG, Zhang H, Tong WP (2016). Superhydrophobic copper stearate/copper oxide thin films by a simple one-step electrochemical process and their corrosion resistance properties. RSC Adv..

[20] Zhang PR, Liu ZQ (2016). Physical-mechanical and electrochemical corrosion behaviors of additively manufactured Cr-Ni-based stainless steel formed by Laser cladding. Mater. Des..

[21] Bender S, Goellner J, Heyn A, Boese E (2007). Corrosion and corrosion testing of magnesium alloys. Mater. Corros..

[22] Kadowaki M, Muto I, Sugawara Y, Doi T, Kawano K, Hara N (2017). Pitting corrosion resistance of martensite of AISI 1045 steel and the beneficial role of interstitial carbon. J. Electrochem. Soc..

[23] Liu ZG, Gao XH, Du LX, Li JP, Kuang Y, Wu B (2015). Corrosion behavior of low-alloy steel with martensite/ferrite microstructure at vapor-saturated CO_2_ and CO_2_-saturated brine conditions. Appl. Surf. Sci..

